# Why Publishing Everything Is More Effective than Selective Publishing of Statistically Significant Results

**DOI:** 10.1371/journal.pone.0084896

**Published:** 2014-01-17

**Authors:** Marcel A. L. M. van Assen, Robbie C. M. van Aert, Michèle B. Nuijten, Jelte M. Wicherts

**Affiliations:** Department of Methodology and Statistics, Tilburg School of Behavioral Sciences, Tilburg University, Tilburg, The Netherlands; State University of New York, United States of America

## Abstract

**Background:**

De Winter and Happee [1] examined whether science based on selective publishing of significant results may be effective in accurate estimation of population effects, and whether this is even more effective than a science in which all results are published (i.e., a science without publication bias). Based on their simulation study they concluded that “selective publishing yields a more accurate meta-analytic estimation of the true effect than publishing everything, (and that) publishing nonreplicable results while placing null results in the file drawer can be beneficial for the scientific collective” (p.4).

**Methods and Findings:**

Using their scenario with a small to medium population effect size, we show that publishing everything is more effective for the scientific collective than selective publishing of significant results. Additionally, we examined a scenario with a null effect, which provides a more dramatic illustration of the superiority of publishing everything over selective publishing.

**Conclusion:**

Publishing everything is more effective than only reporting significant outcomes.

## Introduction

Scientific publications typically report positive rather than negative empirical results [2], [3], suggesting that empirical results in the literature do not form a comprehensive or representative sample of all empirical results. It is widely agreed that publication bias, or the selective reporting of empirical studies on the basis of their statistical significance, may inflate effect sizes estimated by meta-analyses.[4]–[13]. A widely held view is that the optimal solution to counter publication bias is to collect all of the available evidence regardless of whether the results are significant or not. This view is contradicted in a recent paper by de Winter and Happee [1] (henceforth W&H) in which they present the results of a simulation study that was based on the premise that science is self-correcting. W&H concluded on the basis of their simulations that it may be more effective to publish results selectively on the basis of outcomes. We agree with W&H that scientific publication is associated with costs in terms of writing and publishing [14], which should be diminished. However, we disagree with their notion that too many published results lead to what others have coined the “cluttered office phenomenon”, meaning that in an office full of academic papers it is hard to tell the good ones from the bad ones [15]. The reason is that statistical methods like meta-analysis have been developed to synthesize all the available evidence to avoid such a clutter of diverging results. Given the potentially far-reaching implications of W&H's conclusions for the publishing of empirical results in many scientific fields, we here criticize their conclusions. Specifically, we reanalyze their simulation scenario and results, and expand their simulations by including a scenario of a null effect (i.e., with false positive findings).

### De Winter and Happee's two approaches

In their simulation study, W&H compared two approaches to scientific publishing in a scenario involving a hypothetical line of research on a given topic. All of W&H's simulations assumed that one underlying population effect gave rise to the study outcomes (i.e., a fixed effect in the parlance of meta-analysis). In W&H's first approach (“publishing everything”) all results were published regardless of their outcome. In their second approach (“selective publishing”), results were published if they deviated significantly from the earlier evidence as presented in the literature. We remark that the notion that all results are published does not match well with the overwhelming evidence of the file-drawer problem in many academic fields [16]–[21], although it is optimal in the statistical sense that more information is better. On the other hand, the selective publishing approach may be problematic for the accumulation of knowledge because it would impede the publication of corroborating evidence of earlier findings.

The selective publishing approach of W&H assumes that each subsequent study in a line of research (i.e., a study on a given topic) involves a test of the null hypothesis that the population effect equals the summary effect obtained by a meta-analysis on the previously published findings (a so-called cumulative meta-analysis). Findings are only published when they are significantly different from the summary effect on the basis of earlier published results (*α* = .05, two-tailed test). If studies have not yet been published in a research line, the null hypothesis is that there is no effect. W&H's selective publishing has at least three merits. First, the model can explain the Proteus phenomenon reflecting the observation that the first publication on a given topic usually overestimates and the second publication tends to underestimate the effect [1], [22]. Second, W&H show that selective publishing yields an accurate estimate of the population effect already after five or more publications. Third, as a consequence, the selective publishing approach provides a more accurate estimate of the population effect size than an approach wherein each study tests the null hypothesis of no effect and mainly studies with statistically significant results are selected for publication. The latter approach would lead to a systematic overestimation of effect size [1], [5], [6], [23].

W&H evaluated the performance of the selective publishing and publishing everything approaches using a straightforward simulation study that involved one scenario in which the population was normally distributed with mean and standard deviation equal 0.3 and 1, respectively. All primary studies in the scenario had a sample size of 50. W&H repeated the simulation 5,000 times, and each simulation was stopped when 40 studies were published in the selective publishing approach. W&H found that the standard deviation of the cumulative meta-analytic effect across all 5,000 replications as a function of publication number was smaller in the selective publishing than in the publishing everything approach (W&H's Figure 3). More specifically, the standard deviation after 40 publications was .0170 for selective publishing compared to the higher .0222 for publishing everything, and 68 studies were needed in publishing everything for reaching the same accuracy obtained after 40 publications in selective publishing ([1], p. 5). Based on these comparisons W&H concluded that the file drawer effect can be beneficial for the scientific collective.

Here we show that W&H's conclusion is false, i.e., that publishing everything is *more effective* than selective publishing. We use ‘effective’ to encompass statistical considerations (i.e., precision of the estimate of meta-analyses), and cost-benefit and time considerations. First, we demonstrate that publishing everything is more effective, using exactly the same simulation results and scenario as used by W&H [1]. Then we show the superiority of publishing everything over selective publishing in a scenario where the population effect is zero and the researcher's goal is to detect small (Cohen's *d* = .2) or larger effect sizes (*d*>.2).

## Method

### Publishing everything is more effective (I): Re-analysis of W&H's results

Using exactly the same simulation results and scenario as W&H, we present two reasons why publishing everything is more effective than selective publishing. The first reason is that the meta-analytic effect is estimated more precisely (i.e., with a lower standard error) in the publishing everything than in the selective publishing approach. Second, publishing everything is also “cheaper” than selective publishing in terms of cost-benefit and time considerations.

A major problem in the evaluation of the two approaches by W&H is that it is based on a number that is not available to the scientist. W&H compared the two approaches using the *standard deviation* of the meta-analytic effect after 5,000 simulations. However, what is available to the scientist is the *standard error* of *one* meta-analytic effect size estimate. The standard error of the meta-analytic effect is a function of the sampling error of each study (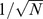), the number of published studies (*K*), and the heterogeneity of the effect as found in the published studies. The underlying effect is homogenous in the publishing everything approach, and the standard error of the meta-analytic effect can be shown to equal 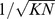. In the selective publishing approach, only studies yielding significantly different effects are published, introducing heterogeneity in effects (see also W&H's Figure 2). This results in a larger standard error of the meta-analytic effect for selective publishing than for publishing everything.

**Figure 1:**
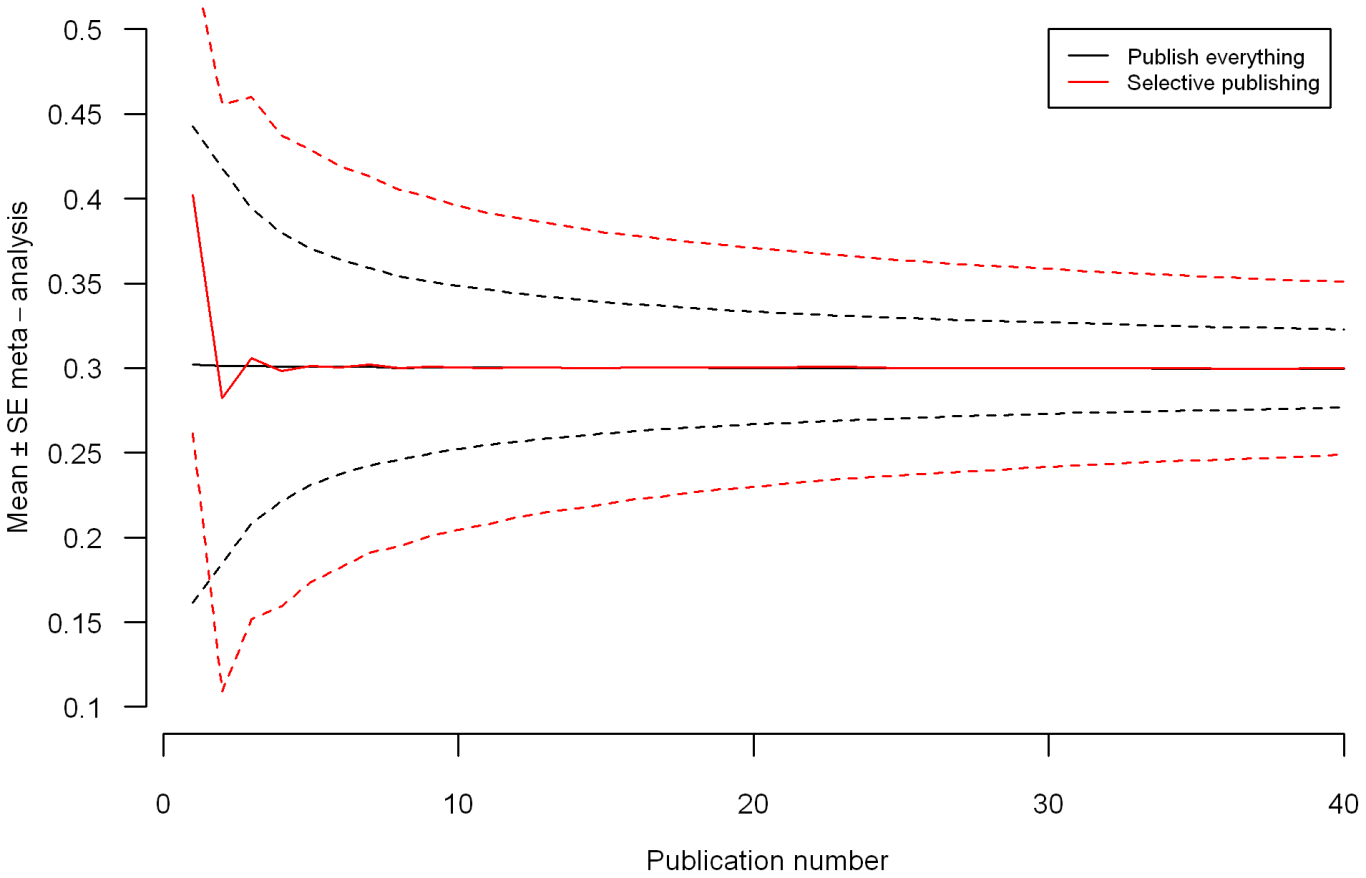
Mean (solid lines) and mean plus/minus one meta-analytic standard error (dashed lines) of estimate obtained by a random-effect meta-analysis as a function of the number of publications for both the publishing everything and selective publishing approaches.

To illustrate the higher precision of the meta-analytic effect in publishing everything, we ran exactly the same simulation as W&H but now recorded the sampling error of the estimate after 40 publications for each of the 5,000 simulations. We applied random-effects meta-analysis, because random-effects meta-analysis is generally recommended when the underlying population effect may be heterogeneous [24]. We used the R package metafor [25] (see Appendix S1 for the R code used in all simulations). Heterogeneity is expressed as the variance 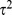 of the underlying effect [24]. Figure 1 shows the cumulative meta-analytic effect ± the standard error in both approaches as a function of publication number. Figure 1, a correction of W&H's Figure 3, mimics the development of the meta-analytic effect but shows that estimation was more precise under the publishing everything approach. The average standard error after 40 publications in the publishing everything approach was 0.023, with 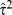 = 0 in 45.6% of the simulations, and an average value of 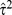 equal to 0.0023. Note that the average standard error is close to both the theoretically derived 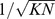 = 0.02236 and the 0.0222 reported by W&H. It can be shown that the expected value of the variance of the meta-analytic effect across simulations is equal to the expected value of the squared standard error of one meta-analysis, if both are calculated under the publish everything approach. The average standard error in the selective publishing approach was 0.051, with 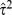
>0 in all simulations and an average value of 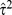 equal to 0.085. Note that the standard error was more than 2.2 times as large in the selective publishing as in the publishing everything approach. To conclude, the estimate of population effect size is more precise under publishing everything than under selective publishing.

The second reason why publishing everything is more effective concerns the neglect of time and money in the analysis of W&H; “… the factor time is not included in our simulation models. That is, results are assessed per publication without taking into account study completion and the time between study completion and publication.” (p2). They compared both approaches conditional on the number of *published* studies. However, the 40^th^ published study in publishing everything corresponds to on average the 704^th^ conducted study (p.3) in the selective publishing approach. Hence on average 17.6 more time evolves and money is spent on data collection and doing research in the selective publishing approach to obtain the same number of 40 published studies in the publishing everything approach. To conclude, publishing everything is also more effective than selective publishing using cost-benefit and time considerations.

### Publishing everything is more effective (II): when effect size matters

In this section, we expand on W&H's results by considering a scenario where the effect size matters. We slightly changed the scenario of W&H, but still compared the same two approaches. Again, the population was normally distributed, standard deviation equaled 1, and each primary study had a sample size of 50. However, the population mean equaled 0 (no effect), rather than 0.3 as in W&H's scenario. Moreover, we introduced a different stopping rule: it was assumed scientists stopped investigating the effect when they rejected the null hypothesis that the population effect is at least small (H_0_: *d* = *μ*≥.2) using the results of the meta-analysis on all published studies. We believe it is safe to assume scientists are no longer interested in studying an effect when it is “smaller than small”. Again we applied random-effects meta-analysis in both approaches.

Table 1 presents the results of the meta-analyses under both approaches. In both approaches the population effect size was estimated accurately as 0 (first row). However, it took on average much less published studies in publishing everything (3.84) than in selective publishing (8.36). Note that a vast number of on average 114 studies was needed to conclude that the effect is ‘smaller than small’ in selective publishing, which is about 30 times as many studies as in publishing everything. Hence this scenario also demonstrates that publishing everything is more effective than selective publishing. Another characteristic of selective publishing is again the heterogeneity in effect sizes (see the rows on 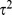). The (absence of) heterogeneity is estimated correctly in publishing everything (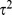 is 0 in 59.4% of the runs, and its average across runs is close to zero), but not in selective publishing (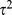 is never zero, and its average corresponds to a considerably large standard deviation of effect size equal to .268). Note that the heterogeneity under selective publishing does not reflect heterogeneity of the underlying population effect size, but is an artifact of selective publishing.

**Table 1:** Results of 5,000 runs of meta-analyses under the publishing everything and selective publishing approaches.

	Publishing Everything	Selective Publishing
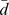 (se)	0.001 (0.083)	0.000 (0.106)
# studies published (sd)	3.84 (1.73)	8.36 (1.64)
# studies conducted (sd)	3.84 (1.73)	113.95 (49.30)
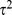 (sd)	0.005 (0.009)	0.072 (0.014)
% 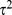 = 0	59.4	0
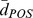 (se)	0.023 (0.082)	0.000 (0.106)
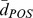 (se)	−0.022 (0.083)	0.000 (0.106)

The final rows of Table 1 present the meta-analytic effect size averaged across all runs having the same sign of the first published study, i.e., negative or positive. One may wonder whether the selective publishing scenario yields a biased estimate of the effect depending on the sign of the first study. The results of our simulation show that meta-analyses with as few as about 8 published studies already yield an unbiased estimate, confirming W&H (p.3) that possible bias in selective publishing (as modeled by W&H) is rapidly nullified.

## Discussion

On the basis of results of their simulation, W&H concluded that, after a fixed number of publications, selective publishing yields a more accurate estimate of the true effect than publishing everything. We argue that their analysis of their results has two weaknesses. First, they use information that a researcher does not have, i.e., the standard deviation of the meta-analytic effect after many simulations; a researcher only possesses a standard error of one meta-analysis on the basis of all available results. Second, W&H analyzed their data disregarding the possible heterogeneity of effect sizes in a given meta-analysis. After re-analyzing their simulation results using random-effects meta-analysis we conclude, contrary to W&H that publishing everything yields a more accurate estimate than selective publishing. We also compared the two approaches in another scenario where the population effect is zero and scientists stop investigating the effect when they reject the null hypothesis that the population effect is at least small. The results in the latter scenario favored publishing everything even more; on average less than 4 studies were needed in the publishing everything approach, whereas more than 8 publications (114 studies) were needed in the selective publishing approach. On top of statistical arguments, we also demonstrate that publishing everything is more efficient than selective publishing with respect to cost-benefit and time considerations.

Here we argued that publishing everything is superior to selective publishing. More generally, in line with what is considered as best practice in meta-analysis, we recommend researchers to include both published *and* unpublished papers [26]. Meta-analyses in W&H's selective publishing approach only consider (published) studies with results that deviate significantly from earlier evidence, and disregard all other (unpublished) studies. A relevant question is what happens to the accuracy of the meta-analytic estimate in selective publishing if a random selection of unpublished studies is included in the meta-analysis in addition to the ones that are published. Assuming all studies estimate the same underlying population effect, the accuracy of the estimate *increases*, since more unbiased information is incorporated in the analysis. Hence, even selective publishing benefits from including unpublished studies. Statistically speaking, more information is always better as long as the information is unbiased. In any case, methods of meta-analysis are needed that yield accurate effect size estimates even under the most extreme scenarios of publication bias [27].

Another relevant question concerns the validity of the selective publishing scenario. It surely has some merit, since it explains the Proteus phenomenon that has occurred in some research areas [1], [22]. We argue, however, that in many research fields studies are most likely to be published when they report effects that are significantly different from zero rather than from the effect estimated in cumulative meta-analysis. The evidence for this type of publication bias is overwhelming. For instance, Fanelli [2], [3] observed a high percentage of ‘positive’ (significant) results in many sciences, particularly in social sciences, e.g. psychology (about 95%, [3], p. 898). This high percentage cannot be explained by studies’ statistical power since power generally is low and there is no evidence that it has grown over the years ([3], p. 899). The high percentage is indicative of publication bias, i.e., the bias introduced by tendencies to mostly submit and publish results that are statistically different from zero.

To the defense of W&H, one may argue that we did not do justice to their fixed effect approach by using random-effects meta-analyses in our simulations. Random-effects meta-analysis assumes a heterogeneous population effect size, but the data in all scenarios are generated from a population with a fixed effect size. However, a researcher is ignorant about the population effect size and cannot a priori determine that there is just one underlying effect in a set of studies. It is standard practice to use random-effects meta-analysis when there is evidence of effect size heterogeneity [24]. Several studies [28], [29], [30] have shown that mistakenly applying fixed-effect meta-analysis to a heterogeneous effect may lead to biased results and erroneous conclusions [31]. Those results also highlighted that applying random-effects meta-analysis when the underlying population effect is fixed does not yield bad results or erroneous conclusions. The problem with the selective publishing scenario is that the researcher does not know whether the data were generated with that scenario or rather that the data reflect true heterogeneity. Fixed-effect meta-analysis is only appropriate if the researcher is certain that the heterogeneity does not reflect heterogeneity in population effect size. If the heterogeneity caused by selective publishing is erroneously interpreted as heterogeneity of the underlying population effect size, then this heterogeneity artifact may lead entire fields astray. Researchers may focus on random aspects of the published studies as potential moderators of the studied effect and interpret them in substantive terms while all they represent is random noise coupled with a selective approach to scientific publishing.

Neither our nor W&H's simulations take into account what we consider major sources of bias in science, namely researcher's own aspirations and expectations in conducting studies, analyzing the data, and reporting of the results. The colloquial notion of “disappointing results” renders actual science notably more complex. Researchers conduct studies with a clear expectation about the results and are not immune to confirmation bias. Moreover, most high-impact journals specifically select for novel results and are commonly believed to select studies predominantly on the basis of statistical significance [32]. The incentives associated with publishing novel and statistically significant research in high-impact journals may lead to strategic behaviors of researchers [33]. Researchers' bias and strategic behavior together with journals' preference for specific results produces published studies that typically overestimate effect size and are likely not a good representation of all conducted studies.

The bias introduced by the contemporary scientific publishing system is quite severe [13], [16], [34], [35] and it is clear that we as scientists need to focus more on getting it right than on getting it published [36]. In research, there are many unknown factors that are hard to comprehend on the basis of only a literature that contains results that deviate significantly from the null hypothesis or from any other hypothesis. This does not mean that using all information is necessarily the most efficient. Publishing results is costly, but setting up and executing studies on the basis of (potentially biased or inaccurate) results in the literature also comes at a major cost that may be much larger than the cost of publishing. What is clearly needed is to lower the bar for publishing replication studies [4], [35], [36]. Although W&H proposed an interesting approach, we argue on the basis of the current results and our philosophical view that rigorous scientific data should never be wasted, and that the preferred approach to publishing is to have all information out there.

## Supporting Information

Appendix S1 **Simulation code (R).**
(R) Click here for additional data file.
